# Unlocking the Constraints of Cyanobacterial Productivity: Acclimations Enabling Ultrafast Growth

**DOI:** 10.1128/mBio.00949-16

**Published:** 2016-07-26

**Authors:** Hans C. Bernstein, Ryan S. McClure, Eric A. Hill, Lye Meng Markillie, William B. Chrisler, Margie F. Romine, Jason E. McDermott, Matthew C. Posewitz, Donald A. Bryant, Allan E. Konopka, James K. Fredrickson, Alexander S. Beliaev

**Affiliations:** a Chemical and Biological Signature Science, Pacific Northwest National Laboratory, Richland, Washington, USA; b Biological Sciences Division, Pacific Northwest National Laboratory, Richland, Washington, USA; c Environmental Molecular Sciences Laboratory, Pacific Northwest National Laboratory, Richland, Washington, USA; d Department of Chemistry and Geochemistry, Colorado School of Mines, Golden, Colorado, USA; e Department of Biochemistry and Molecular Biology, The Pennsylvania State University, University Park, Pennsylvania, USA; f Department of Chemistry and Biochemistry, Montana State University, Bozeman, Montana, USA; g Department of Biological Sciences, Purdue University, West Lafayette, Indiana, USA; h The Gene and Linda Voiland School of Chemical Engineering and Bioengineering, Washington State University, Pullman, Washington, USA

## Abstract

Harnessing the metabolic potential of photosynthetic microbes for next-generation biotechnology objectives requires detailed scientific understanding of the physiological constraints and regulatory controls affecting carbon partitioning between biomass, metabolite storage pools, and bioproduct synthesis. We dissected the cellular mechanisms underlying the remarkable physiological robustness of the euryhaline unicellular cyanobacterium *Synechococcus* sp. strain PCC 7002 (*Synechococcus* 7002) and identify key mechanisms that allow cyanobacteria to achieve unprecedented photoautotrophic productivities (~2.5-h doubling time). Ultrafast growth of *Synechococcus* 7002 was supported by high rates of photosynthetic electron transfer and linked to significantly elevated transcription of precursor biosynthesis and protein translation machinery. Notably, no growth or photosynthesis inhibition signatures were observed under any of the tested experimental conditions. Finally, the ultrafast growth in *Synechococcus* 7002 was also linked to a 300% expansion of average cell volume. We hypothesize that this cellular adaptation is required at high irradiances to support higher cell division rates and reduce deleterious effects, corresponding to high light, through increased carbon and reductant sequestration.

## IMPORTANCE

Efficient coupling between photosynthesis and productivity is central to the development of biotechnology based on solar energy. Therefore, understanding the factors constraining maximum rates of carbon processing is necessary to identify regulatory mechanisms and devise strategies to overcome productivity constraints. Here, we interrogate the molecular mechanisms that operate at a systems level to allow cyanobacteria to achieve ultrafast growth. This was done by considering growth and photosynthetic kinetics with global transcription patterns. We have delineated putative biological principles that allow unicellular cyanobacteria to achieve ultrahigh growth rates through photophysiological acclimation and effective management of cellular resource under different growth regimes.

## INTRODUCTION

It has long been established that photoautotrophic growth is dependent on the combined rates of light, carbon, and macronutrient acquisition and the efficiencies by which these resources are directed toward biomass synthesis (1). The inherent differences between the rates of light and dark processes highlight an important paradigm that defines cyanobacterial productivity constraints, whereby photosynthetic energy acquisition and CO_2_ fixation are interdependent but maintain distinct enzymatic mechanisms and kinetics (2, 3). Under high irradiances, photosynthesis is adversely affected by damage (photoinhibition) to the light-harvesting machinery (1) and/or by photorespiration as RuBisCO selectivity shifts toward O_2_ (4). Notably, most of what is known about these effects as a function of irradiance and O_2_ tension concerns photosynthesis (5–7) rather than cell growth.

The typical range of doubling times for well-characterized unicellular cyanobacteria (e.g., *Synechocystis* sp. strain PCC 6803 and *Synechococcus elongatus* PCC 7942) is between 7 and 12 h (8, 9). Several hypotheses concerning mechanisms constraining cyanobacterial growth rates have been proposed; these include spatial restrictions within the cell that limit diffusion processes (10) as well as metabolic costs that determine partitioning of cellular resources and resulting fitness (11, 12). The optimization model, developed to simulate the partitioning of material and energy within a photoautotrophic cell (11), has extended the concept of growth as a function of proteome allocation between adaptation to niche-specific environments and cell division resources. An important implication of this is the ability to minimize the requirement for niche-adaptive responses that may be a key for cyanobacteria to redirect energy and nutrients efficiently toward the biosynthesis of biomass (13). This also holds significance for understanding regulatory switches governing central metabolism and secondary biosynthetic pathways, which are primary targets for bioengineering of cyanobacteria (14, 15).

In this study, we systematically dissected the growth and photophysiological performance of *Synechococcus* sp. strain PCC 7002 (hereafter *Synechococcus* 7002), a fast-growing euryhaline unicellular cyanobacterium (16) that has become a promising biotechnological platform (17–20). Although the physiological behavior of *Synechococcus* 7002 has been investigated under a wide range of irradiance, temperature, and salinity conditions (1, 9, 21–23), there is still a paucity of information concerning principles underlying cyanobacterial growth efficiency and robustness. The photosynthetic potential, thresholds of inhibition, and growth are not explicitly equivalent, and significant variation is expected between different environments (24). Herein, we compared the growth and photosynthetic rates of *Synechococcus* 7002 across varied incident irradiance and dissolved O_2_ with a slower growing unicellular cyanobacterium, *Cyanothece* sp. strain ATCC 51142 (hereafter *Cyanothece* 51142) (25). *Cyanothece* 51142 was chosen as a comparative organism because it is a well-characterized strain that is amenable to continuous cultivation (15, 17, 26). The growth rates of *Cyanothece* 51142 are similar, compared to those displayed by other model cyanobacterial strains, such as *Synechocystis* strain 6803, and *Synechococcus elongatus* strain 7942 (8, 9). The irradiance-dependent response was further investigated through global RNA sequencing analysis to correlate the ultrafast growth with the photophysiological dynamics and gene expression of *Synechococcus* 7002. Through integration of state-of-the-art cultivation with photophysiological kinetic analyses and transcriptomic measurements, this genome study provides a new level of insight into the mechanisms guiding the energy and resource partitioning in cyanobacteria and sheds light on the phenomenon of ultrafast photoautotrophic growth.

## RESULTS

### Ultrafast growth under high irradiance.

**FIG 1:**
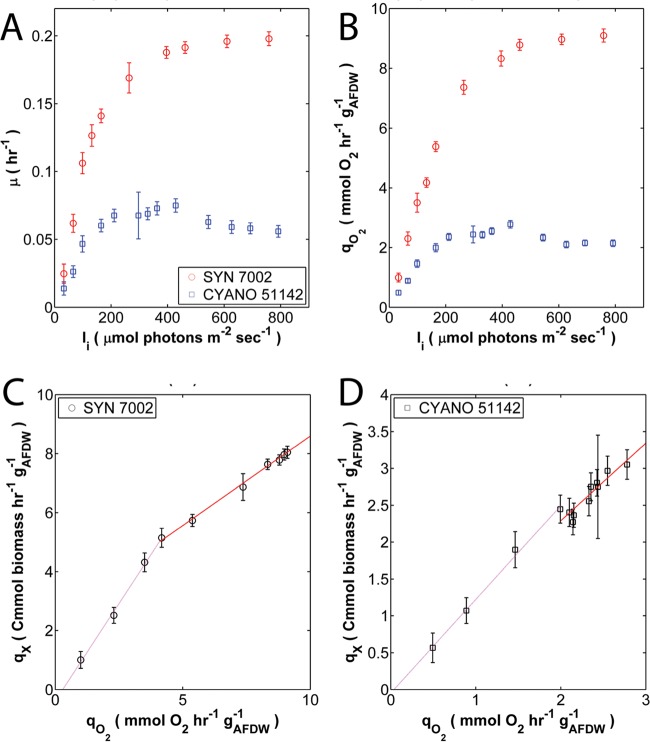
Steady-state growth and photosynthesis values of *Synechococcus* 7002 and *Cyanothece* 51142 controlled at different incident irradiances. (A) Specific growth rates of *Synechococcus* 7002 (SYN 7002) (red circles) and *Cyanothece* 51142 (CYANO 51142) (blue squares). (B) Net specific O_2_ production rates (i.e., net oxygenic photosynthesis) of *Synechococcus* 7002 and *Cyanothece* 51142. (C and D) Net specific rates of biomass production on a carbon mole basis (*q_X_*) plotted against the net oxygenic photosynthesis (*q*_O2_) for *Synechococcus* 7002 (C) and *Cyanothece* 51142 (D). The slopes represent the net growth-to-photosynthesis yield (Q) corresponding to either the light-limited phase (pink line) or the light-saturated phase (red line). Values are means ± 1 standard deviation (error bars).

Optically thin (optical density at 730 nm [OD_730_] of 0.082 ± 0.003) cultures of *Synechococcus* 7002 and *Cyanothece* 51142 were grown under turbidostat control to maintain nutrient-replete steady states operating at the maximum specific growth rate obtainable for a given environmental condition (27, 28). Under these conditions, *Synechococcus* 7002 displayed specific growth rates (μ) that increased with irradiance to a maximum value of 0.20 ± 0.01 h^−1^. Growth was not inhibited even at the highest incident irradiance (*I_i_*), 760 µmol photons ⋅ m^−2^ ⋅ s^−1^, examined in this study (Fig. 1A). In contrast, specific growth rates for *Cyanothece* 51142 reached a maximum of 0.07 ± 0.01 h^−1^ at 430 µmol photons ⋅ m^−2^ ⋅ s^−1^ and then declined at higher irradiances. The μ-*I_i_* relationships displayed saturating and/or peak functional trends similar to typical photosynthesis-to-irradiance (*P-I*) curves (see Fig. S1 in the supplemental material) (29, 30).

The steady-state net specific rates of photosynthesis (*q*_O2_) followed a similar increase as μ (Fig. 1B). Net O_2_ production by *Synechococcus* 7002 reached and sustained a maximum value of 9.1 ± 0.2 mmol O_2_ ⋅ h^−1^ ⋅ g_AFDW_^−1^ (AFDW stands for ash-free weight [dry weight]), while *Cyanothece* 51142 cultures peaked at 2.8 ± 0.1 mmol O_2_ ⋅ h^−1^ ⋅ g_AFDW_^−1^ and subsequently decreased at higher irradiances. These results confirm that unlike *Synechococcus* 7002, *Cyanothece* 51142 is susceptible to photoinhibition during steady-state growth at *I_i_* of >430 µmol photons ⋅ m^−2^ ⋅ s^−1^. The observed effects on growth occurred at low dissolved O_2_ concentrations (≤8.0 and ≤2.5 µM for *Synechococcus* 7002 and *Cyanothece* 51142, respectively).

Based on the growth and photosynthetic relationships with *I_i_*, the physiological responses can be categorized into two distinguishable regimes: (i) a “light-limited” regime, characterized by an increasing linear response to *I_i_*; and (ii) a “light-saturated” regime, which varied from asymptotic growth in *Synechococcus* 7002 to inhibition in *Cyanothece* 51142. The two regimes were separated by a “transitional” phase defined here as the responses at or near the theoretical saturating irradiance *I_k_* (31). The observed bimodal behavior was further supported by net growth-to-photosynthesis yields that are proxies for the photosynthetic quotient (Q) (Cmmol biomass/mmol of O_2_), which was defined here as the ratio of the net rate of carbon fixation into biomass to the net rate of oxygenic photosynthesis. For *Synechococcus* 7002, the photosynthetic quotients were higher during light-limited growth (Q_lim_ = 1.3 ± 0.3) than light-saturated growth (Q_sat_ = 0.6 ± 0.1) (Fig. 1C). In contrast, the Q values calculated for growth of *Cyanothece* 51142 varied only from 1.3 ± 0.2 to 1.1 ± 0.4 during light-limited and light-saturated conditions, respectively (Fig. 1D). These results show that Q is greatest during light-limited growth of *Synechococcus* 7002 and also indicate that more reductant is required to support fast growth at higher incident irradiances. The same cannot be concluded for *Cyanothece* 51142, as much slower (≥3-fold) growth rates are supported by an effectively constant amount of net oxygenic photosynthesis regardless of whether *I_i_* is limiting or saturating (i.e., inhibiting) relative to growth.

### Photosynthetic performance as a function of growth phase and irradiance.

**FIG 2:**
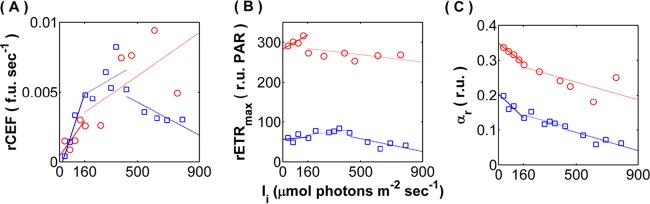
Photosynthesis parameters determined by chlorophyll fluorescence methods. (A) Surrogate rate of cyclic electron flow (rCEF); (B) relative maximum electron transport rate (rETR_max_); (C) relative maximal quantum yield of photochemistry (α_r_). Data points specific to *Synechococcus* 7002 and *Cyanothece* 51142 are represented by red circles and blue squares, respectively. Linear regression was used to establish positive or negative trends of each parameter during light-limited growth (solid lines), saturated/peak growth (dotted lines), and photoinhibited growth (dashed lines). f.u., fluorescence units; r.u., relative units.

Interrogation of the photosynthetic apparatus via chlorophyll (chlorophyll *a* [Chl *a*]) fluorescence techniques identified further differences between how *Synechococcus* 7002 and *Cyanothece* 51142 acclimate to increasing irradiance. As a general trend, the parameters associated with photosystem performance and photosynthetic electron transfer demonstrated distinct bimodal distributions as a function of light availability (i.e., limited versus saturated regimes). The most remarkable difference between *Synechococcus* 7002 and *Cyanothece* 51142 was the contrasting trends of cyclic electron flow (rate of cyclic electron transport [rCEF]), determined by postillumination fluorescence (Fig. 2A). *Synechococcus* 7002 displayed a positive rCEF rate trend across all *I_i_* values, with the exception of the highest illumination (760 µmol photons ⋅ m^−2^ ⋅ s^−1^). In contrast, the rCEF rates in *Cyanothece* 51142 followed both growth and photosynthetic trends and displayed three distinct phases: a fast increase in rCEF associated with light-limited growth (*I_i_* < 430 µmol photons ⋅ m^−2^ ⋅ s^−1^), deceleration during the transition *I_k_* (~430 µmol photons ⋅ m^−2^ ⋅ s^−1^), and steep decline under light-saturated conditions (*I_i_* > 430 µmol photons ⋅ m^−2^ ⋅ s^−1^) associated with inhibition of growth and photosynthesis.

The maximum relative rate of electron transport (rETR_max_) (Fig. 2B) shifted from a positive trend to a negative trend at the transition from light-limited to light-saturated growth for both cyanobacteria. This result indicates that the photosynthetic potential increased with increasing growth under light-limited regimes but leveled off and even decreased under light-saturated growth. Notably, the rETR_max_ values measured in *Synechococcus* 7002 were approximately 3-fold higher than those displayed by *Cyanothece* 51142. Furthermore, the relative maximal quantum yield of photochemistry (α_r_) was the highest during light-limited growth and decreased with increasing *I_i_* for both cyanobacteria (Fig. 2C). It should be noted that some of the photosynthesis parameters measured for *Synechococcus* 7002 (e.g., rCEF and α_r_) during the steady state corresponding to the highest *I_i_* (760 µmol photons ⋅ m^−2^ ⋅ s^−1^) deviated from the predominant trends observed during light-saturated growth (see Fig. S2 in the supplemental material).

### Growth tolerance to increasing O_2_ tension.

**FIG 3:**
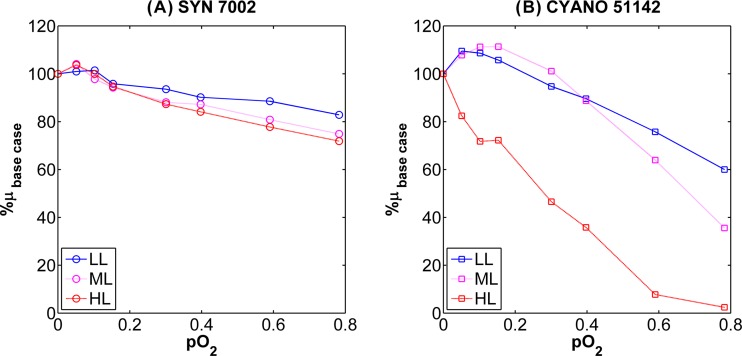
Relative responses of specific growth rate to combinatorial increases in oxygen tension (i.e., partial pressures, pO_2_) and incident irradiance. Data are represented as the percent decrease from the baseline growth obtained by sparging the cultures with 2% CO_2_ in N_2_. (A) *Synechococcus* 7002 oxygen stress profile containing low-light (LL), medium-light (ML), and high-light (HL) conditions corresponding to incident irradiance values of 99, 132, and 760 µmol photons m^−2^ s^−1^. (B) *Cyanothece* 51142 oxygen stress profile reporting LL, ML, and HL conditions corresponding to incident irradiance values of 131, 395, and 790 µmol photons m^−2^ s^−1^.

To further define the boundaries of cyanobacterial growth robustness and to simulate the effects of elevated O_2_ tensions that can occur under intense photosynthetic activity, the steady-state turbidostat-grown cultures were subjected to increasing partial O_2_ pressure (pO_2_). While growth of both *Synechococcus* 7002 (Fig. 3A) and *Cyanothece* 51142 (Fig. 3B) was inhibited by O_2_ to a greater degree under high irradiance relative to low irradiance, there were stark differences between the sensitivities of the two organisms to elevated pO_2_. *Synechococcus* 7002 was much more resistant to the increasing pO_2_ levels, with only a 28% decline in growth rate at a pO_2_ of 0.78 (840 µM) and *I_i_* of 760 µmol photons ⋅ m^−2^ ⋅ s^−1^. In contrast, the growth rates of *Cyanothece* 51142 decreased severely with increasing O_2_ tension under the same irradiance. At a pO_2_ of 0.78 and *I_i_* of 760 µmol photons ⋅ m^−2^ ⋅ s^−1^, growth of *Cyanothece* 51142 was nearly completely (~98%) inhibited. Interestingly, growth rates for *Cyanothece* 51142 increased in response to increasing pO_2_ values up to 0.1 (107 µM), but only at irradiances of <400 µmol photons ⋅ m^−2^ ⋅ s^−1^.

### Transcriptional responses reveal potential mechanisms of growth robustness.

**FIG 4:**
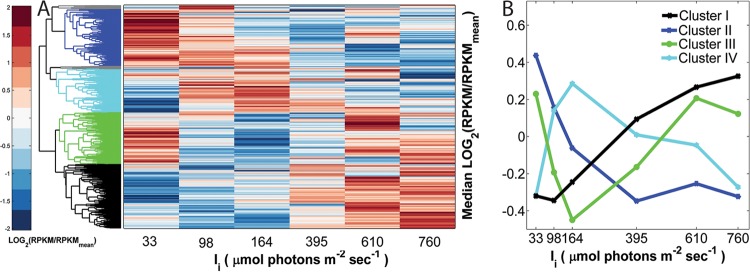
Hierarchical clustering of relative transcript abundances in *Synechococcus* 7002 during steady-state turbidostat growth as a function of incident irradiance. The major clusters of coexpressed genes are color coded as follows. Cluster I genes are shown in black (809 genes), cluster II genes are shown in dark blue (724 genes), cluster III genes are shown in green (648 genes), and cluster IV genes are shown in light blue (551 genes). The full 2,732-gene list of the relative expression values at each steady state is given in Table S1 in the supplemental material. (B) The eigenvector profiles of the four main clusters of differentially expressed genes. The same color coding used for panel A is used for panel B.

**FIG 5:**
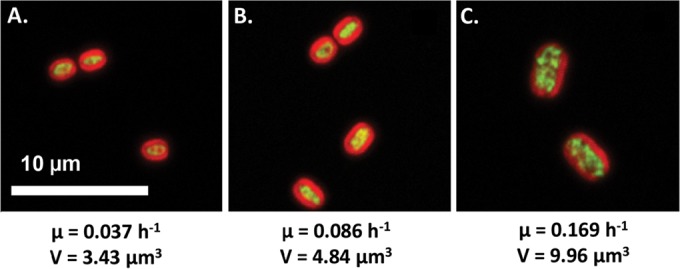
Increase in average cell volumes of *Synechococcus* 7002 cells as a function of irradiance-driven specific growth rates. The volumes were calculated for steady-state cultures grown at *I_i_* values of 66 (A), 98 (B), and 395 (C) µmol photons ⋅ m^−2^ ⋅ s^−1^ and represent an average of 104, 107, and 65 individual cell measurements, respectively. The cells were visualized by autofluorescence (red) and SYBR gold (green). Micrograph images representative of each condition were standardized by the bar.

The *Synechococcus* 7002 gene-level responses to light-controlled growth were further interrogated by RNA sequencing. Hierarchical clustering of relative transcript abundances (reads per kilobase per million reads [RPKM]) across a 2,732-gene data set identified four major clusters, whose eigengenes aligned with the growth and photosynthetic performance of *Synechococcus* 7002 across the irradiance scale (Fig. 4; see Table S1 in the supplemental material). Of the four clusters, cluster I contained the largest group of transcripts (29.6%), the relative abundance of which increased in direct proportion with growth rate. Cluster I was functionally enriched (*P* < 0.05) for genes involved in translation (i.e., ribosomal proteins and tRNA aminoacylation), purine biosynthesis, iron-sulfur cluster assembly, and ATP synthesis (Table 1; Table S2). In concert with the putative upregulation of growth-related functions, there was also a broad increase in transcripts involved in amino acid biosynthesis, protein folding, and iron transport and acquisition. Cluster I also contained genes involved in central carbon metabolism reactions such as the anaplerotic pathways containing bifunctional fructose-1,6-bisphosphatase-II/sedoheptulose-bisphosphatase regulated by photosystem I (PS I) activity (32). Furthermore, reflecting an increased demand for CO_2_ as an electron sink, the relative mRNA levels of genes of the NADH-plastoquinone oxidoreductase complex involved in CO_2_ uptake (*ndhD3*, *ndhF3*, and *cupS*) (33) were elevated in response to increasing irradiance. While not displaying significant functional enrichment within cluster I, the relative transcript abundance of genes involved in peptidoglycan biosynthesis (*mreB* and *mreC*) and cell division (*minCDE*) increased with growth rate. Remarkably, confocal microscopic analysis of *Synechococcus* 7002 turbidostat cultures revealed that the average cell volume changed proportionally with growth rate (Fig. 5). Specifically, the transition of *Synechococcus* 7002 from light-limited (*I_i_* = 98 µmol photons ⋅ m^−2^ ⋅ s^−1^) to light-saturated growth (*I_i_* = 395 µmol photons ⋅ m^−2^ ⋅ s^−1^) resulted in doubling of the average cell volume from 4.84 to 9.69 µm^3^.

**TABLE 1:** Functional enrichment of irradiance-regulated genes of *Synechococcus* 7002

Role or system and cluster	Functional category	% genes^a^	Ratio^b^	*P* value^c^
Main role				
I	Translation	13.1	2.5	4.36E−13
I	Amino acid metabolism	5.6	1.8	1.33E−03
II	Cell motility and adherence	1.7	2.6	1.01E−02
IV	Glycan biosynthesis and metabolism	3.8	1.7	3.43E−02
Subrole				
I	Ribosomal proteins: synthesis and modification	6.8	3.1	1.27E−09
I	tRNA biogenesis	2.5	3.3	1.73E−04
I	Iron-sulfur clusters	0.9	3.5	1.89E−02
II	Peptidases	4.0	1.8	1.28E−02
II	Photosynthesis-antenna proteins	1.7	2.2	2.94E−02
II	Surface structures and assembly platforms	1.4	2.5	2.64E−02
III	Plasmid functions	1.4	2.2	4.82E−02
III	RNA degradation	1.2	2.6	4.31E−02
III	Protein modification and repair	0.9	3.0	3.95E−02
III	Riboflavin metabolism	0.8	3.5	3.78E−02
IV	Toxin-antitoxin systems	4.9	1.7	2.54E−02
IV	Two-component systems	3.4	1.7	4.09E−02
IV	Polysacharide and lipopolysaccharide metabolism	2.7	2.4	7.89E−03
Subsystem				
I	Ribosome large subunit	3.7	3.7	5.13E−07
I	Ribosome small subunit	2.3	3.6	9.03E−05
I	tRNA aminoacylation	2.3	3.6	9.03E−05
I	Purine biosynthesis from ribose-5-phosphate	1.1	2.7	2.80E−02
I	F_1_/F_o_ ATPase AtpABCDEFGH	1.1	3.6	7.02E−03
I	Iron-sulfur cluster assembly SUF system	0.7	3.4	3.13E−02
II	Photosystem II main subunits	1.0	3.4	1.74E−02
II	Photosystem I other common subunits	0.6	4.4	4.32E−02
II	P-type pilus	0.6	4.4	4.32E−02
III	NAD(P)H:quinone oxidoreductase (complex I)	1.9	2.5	1.31E−02
III	High-affinity urea uptake system UrtABCDE	0.6	3.9	4.97E−02
IV	Polysaccharide biosynthesis	3.1	2.9	9.04E−04
IV	Polymorphic toxins	1.8	2.2	3.37E−02
IV	Nickel-dependent hydrogenase	1.8	4.5	8.20E−04
IV	N-type Na translocation ATPase	1.3	4.5	4.91E−03
IV	Glycolipid biosynthesis	0.9	4.8	1.45E−02
IV	Light-independent protochlorophyllide reductase	0.7	5.8	1.98E−02
IV	T2bSS type IVa pilus complex	0.7	3.9	4.67E−02
IV	Pentose phosphate pathway oxidative phase	0.5	5.8	4.49E−02
IV	Polyphosphate kinase/exopolyphosphatase system	0.5	5.8	4.49E−02

a The percentage of genes of a given function within a given functional category.

b The percentage of a particular functional category within the cluster versus the percentage of genes of that functional category within the genome as a whole.

c The *P* value represents the probability that the number of genes associated with a specific pathway/regulon occurs by chance.

Gene expression levels in cluster II, which contained 26% of the total transcripts, were inversely correlated with the growth rate (Fig. 4 and Table 1; see Table S3 in the supplemental material). This cluster was functionally enriched in genes encoding photosystems I and II antenna proteins and contained most of the critical genes required for photosynthesis. Among other transcripts were those encoding key putative signal transduction and regulatory proteins such as the thioredoxin-responsive regulator of photosynthesis and carbon fixation (*pedR*), the ferric uptake regulator (*fur*), and the positive phototaxis signal transduction system (*pixL*-*pixGH*), which was downregulated in excess light. Notably, the increase in specific growth rates was associated with a relative decline in transcript abundance of *Synechococcus* 7002 group 2 and 3 RNA polymerase σ factors, which are typically required for the expression of stress-induced pathways or niche-adaptive protein functions (34).

The eigengene profiles of clusters III and IV exhibited minima and maxima, respectively, at the steady state associated with the transition from light-limited to light-saturated growth (*I_k_* of ≈164 µmol photons ⋅ m^−2^ ⋅ ^−1^). Cluster III (Fig. 4 and Table 1; see Table S4 in the supplemental material), which contained 23% of the expressed genome, included genes associated with photodamage and stress responses, including PS I and PS II stability and repair. Among the genes displaying increased relative mRNA abundance during light-saturated growth were two carboxysome structural genes (*ccmK2* and *ccmK4*), the carbonic anhydrase gene (*ccaA*), the RuBisCO subunits (*rbcSL*), and the CO_2_ fixation transcriptional regulator (*ccmR*). The genes encoding the core subunits of the Ndh-1 complex of *Synechococcus* 7002 (*ndhHIJK* and *ndhA-ndhB-ndhCG*) along with the NdhD1 subunit responsible for cyclic electron transfer around PS I also showed irradiance-dependent increases under light-saturating conditions (33). Cluster IV, containing 20.2% of the transcriptome, exhibited gene expression trends inverse to cluster III; its eigengene displayed increase under light-limiting conditions and then decreased for each successive steady state within the light-saturated regime (Fig. 4, Table 1, and Table S5). Cluster IV was functionally enriched for polysaccharide and glycolipid biosynthesis, the oxidative branch of the pentose phosphate pathway, nickel-iron hydrogenase biogenesis, and genes involved in electron transport processes such as cyclic electron flow at low light intensities (*psaE*) (35) and oxidative phosphorylation (*coxAB* and *sucC*). While many of these pathways can serve as potential reductant sinks (36), they were downregulated under light-saturating conditions, suggesting the presence of alternative mechanisms of reductant partitioning and oxidative stress mitigation in *Synechococcus* 7002.

## DISCUSSION

This study yields insight into an important biological principle, which allows unicellular cyanobacteria to achieve ultrafast growth by having ultrahigh growth rates and manage cellular resource under different irradiance-controlled growth regimes. Herein, we provide direct evidence that the bimodal transition around the theoretical saturating irradiance (*I_k_*) extends not only to the adjustment of the photosynthesis and growth rates but is also coupled to regulation of specific metabolic reactions and cellular functions. The mechanisms by which *Synechococcus* 7002 mitigates the negative effects of high irradiance that typically inhibit the growth and photosynthesis processes of unicellular cyanobacteria are likely to have broader implications for understanding the metabolic and regulatory underpinnings of photosynthetic growth (2, 3).

Consistent increases in relative transcript abundance with increasing irradiance was observed for genes encoding translational machinery, amino acid and nucleotide biosynthesis, the ATPase complex, and the anaplerotic pathways of central carbon metabolism (Fig. 4, cluster I; see Table S2 in the supplemental material). At the same time, the irradiance-driven increase in the relative growth rate of *Synechococcus* 7002 coincided with the broad decrease in transcripts encoding light acquisition machinery and photosystem I and II reaction centers. These coupled transcriptional responses suggest that the level of resources expended for biomass (i.e., protein synthesis) and energy (ATP) synthesis continue to increase with irradiance, while biomass production and net photosynthesis rates are essentially constant due to increased resource expenditure required.

Furthermore, our data provide direct experimental support to earlier calculations (11) positing that energy demand increases to sustain growth, even when growth is light saturated, across increasing irradiance inputs. Reduced net growth-to-photosynthesis yields (Q_lim_ > Q_sat_) during light-replete steady states (Fig. 2) confirm that, while *Synechococcus* 7002 growth is not inhibited at the high-*I_i_* treatments, the ratio of biomass production to energy acquisition decreases once irradiance exceeds an optimum observed near *I_k_*. This is most likely due to the decrease in the optical cross section of the photosynthetic apparatus, as *Synechococcus* 7002 transitions from light-limited to light-saturated growth (1). This transition has negative effects upon the quantum efficiencies of PS II, and in other oxygenic phototrophs, it is linked to increased photoinhibition. Here, we suggest that the reduction in antenna size also allows the cells to redirect carbon and energy fluxes toward biosynthetic processes which fuel cell division. Notably, this metabolic redistribution in *Synechococcus* 7002 occurs in conjunction with a tripling in average cell volume (Fig. 5), a physiological phenomenon that is known to also occur in heterotrophic organisms (i.e., *Salmonella enterica* serotype Typhimurium) in response to increased rate of division (37). These increases in cell mass and size are thought to accommodate the changes in the number of nuclei/cell and can relieve molecular crowding that limits cell growth (11). To that end, removing physical constraints may increase the intracellular capacity needed to accommodate the biosynthetic machinery supporting higher growth rates and alleviate photoinhibition through increased reductant sequestration capacities.

This mechanistic concept is further corroborated by the absence of observed photoinhibition within the *Synechococcus* 7002 steady states (Fig. 3), as increases in irradiance correlated with the generally decreased mRNA levels of known light-sensitive photosystem reaction centers (*psbAD* and *psaAB*). Interestingly, *Synechococcus* 7002 may be unusual, as the lack of elevated transcription of *psb* genes under increasing irradiance contrasts with other transcriptional studies of various slower-growing cyanobacteria exposed to differential and/or stress-inducing light regimes (38–42). The signatures of PS II inhibition were also not observed in the Chl *a* fluorescence analyses performed on *Synechococcus* 7002 (data not shown). For example, the relative maximum rate of electron transport (rETR_max_) increased through the light-limited regime and showed no significant change during light-saturated steady-state growth (transitional saturated states). Furthermore, the regulation of genes mediating reactive oxygen species (ROS) scavenging does not occur uniformly at the transcriptional level. This is consistent with previous gene expression studies (23, 43, 44), which suggested that lack of strong concerted upregulation of stress response machinery under high irradiance levels may reflect other mechanisms employed by *Synechococcus* 7002 for dealing with excess reductant to avoid photoinhibition.

A key observation in support of the above conclusion is based on substantially elevated levels of rCEF displayed by *Synechococcus* 7002 under saturating high irradiances (Fig. 2), indicating reduction of the plastoquinone (PQ) pool from electron donors not associated with PS II (22). While the exact mechanisms and role of CEF are not fully understood in cyanobacteria, it is postulated that this process contributes to balancing reductant and ATP pools, especially under low irradiance levels (45). Interestingly, *psaE* and *ndhOP* genes, encoding the PS I subunit and PQ-oxidoreductase, respectively, which were previously implicated in CEF (35, 46), displayed maximum relative expression only during the transitional steady state but were downregulated at saturating irradiances (cluster IV in Table S5 in the supplemental material). In contrast, genes encoding the core subunits of the Ndh-1 complex along with the NdhD1 subunit responsible for cyclic electron transfer around PS I (*ndhHIJK* and *ndhA-ndhB-ndhCG*) showed irradiance-dependent increase in abundance under light-saturating conditions (cluster III in Table S4). Thus, our data implicate new genes (i.e., *ndhHIJK* and *ndhA-ndhB-ndhCG*) with potentially important roles in the cyclic electron transport of *Synechococcus* 7002 that are inherently linked to its ability to effectively partition reductant fluxes and avoid detrimental effects of oxidative stress using multiple strategies.

In summary, a coordinated, functionally grouped gene expression was observed which broadly supports inferences about the role that transcriptional regulation may play in processes such as photosynthesis, carbon fixation, electron transport, and stress response. Interestingly, the kinetics of growth and photosynthesis revealed bimodal growth regimes with respect to carbon uptake to chemical energy production yields, providing evidence of increased cellular energy expenditure during growth at high (but not inhibiting) irradiances. This effect was corroborated by observations that the relative expression of genes involved in biomass synthesis and chemical energy production continued to increase even when the growth and photosynthetic rates were essentially constant, showing strong evidence for an increased energy requirement with increasing light energy input. Only a few other studies have ever demonstrated distinct saturating and/or inhibition kinetics of specific growth rate as a function of irradiance (47–50). Finally, this study sets a bench mark for specific growth rate (μ = 0.2 h^−1^) achieved by mesophilic cyanobacteria (16, 43, 51) and provides a foundation for understanding the linkages between photosynthetic performance and growth which can bring fundamental new insights that are broadly applicable to other photosynthetic and nonphotosynthetic biological systems.

## MATERIALS AND METHODS

### Bacterial cultures and growth.

*Synechococcus* sp. strain PCC 7002 and *Cyanothece* sp. strain ATCC 51142 were cultured in modified A-plus medium which contained 17 mM NH_4_Cl (52). Starter cultures for the bioreactor were initiated from frozen stocks and were grown as batch cultures in sealed serum bottles charged with growth media supplemented to 15 mM NaHCO_3_ under 50 µmol photons ⋅ m^−2^ ⋅ s^−1^ white-light illumination (tungsten incandescent “soft white”). Controlled cultivation was performed using the New Brunswick Bioflo 310 fermentor platform (Eppendorf, Inc., Enfield, CT) equipped with previously described custom-built light-emitting diode (LED) photobioreactor (28). Photobioreactors were operated at a 5.5-liter volume with 250 rpm agitation and maintained at 30°C and pH 7.5 (controlled via 2 M NaOH and HCl additions). Scalar incident irradiance (*I_i_*) ranged from 33 to 759 µmol photons ⋅ m^−2^ ⋅ s^−1^ and combined 680- and 630-nm narrow-band spectra from LED illuminator chips (Marubeni America Corporation, New York, NY). The reactor was sparged at a constant 4.1 liters min^−1^. In-gas composition was set as a partial nitrogen pressure (pN_2_) of 0.98 and a pCO_2_ of 0.02 during typical growth conditions; however, O_2_ stress conditions were tested by modulating pO_2_ from 0 to 0.8 and decreasing pN_2_ accordingly. Steady-state biomass concentrations were measured directly as ash-free weight (dry weight) (AFDW) (milligram/liter AFDW) (53) and compared in a standard curve to the indirect OD_730_ measurements obtained using a Genesys 20 visible spectrophotometer (Thermo Scientific, Rockford, IL). Dissolved O_2_ concentrations in the reactor was measured with a Clark type O_2_ electrode (InPro 6800 series; Mettler Toledo International Inc., Columbus, OH). Physiological steady state was inferred from continuity (≤3% variation between measurements) of the following growth readouts: OD_730_, pH, and dissolved O_2_ concentration. Samples for all the analyses were taken after at least five residence times at steady-state conditions.

### Turbidostat control.

For turbidostat operation, constant optical densities (OD_730_) of 0.082 ± 0.003 (36 ± 1 mg_AFDW_ liter^−1^) were maintained for both cultures across the entire range of irradiances to effectively eliminate self-shading. Incident and transmitted irradiance was measured with six opposing 2π quantum sensors (LI-210SA photometric sensor; LI-COR Biosciences, Lincoln, NE) and intercalibrated with a 4π submerged quantum sensor (LI-193SA spherical underwater quantum sensor; LI-COR Biosciences). Hence, scalar incident irradiance (*I_i_*) is reported here as quanta incident to the center of the reactor and has been confirmed to be both axially and radially isotropic within the liquid culture volume. *In situ* photosynthesis-to-irradiance curves (*P*-*I_i_* curves) were generated by temporarily stopping flow and subsequently increasing the *I_i_* output of the LEDs over 5-min intervals while concurrently data logging the dissolved O_2_ (DO) response within the culture volume (see photosynthesis calculations below).

### Microscopic analysis.

Microscopic images were acquired on a Zeiss LSM 710 scanning confocal laser microscope (Carl Zeiss MicroImaging GmbH, Jena, Germany) equipped with a W Plan-Apochromatic 63×/1.0 M27 objective. The *Synechococcus* 7002 cells were visualized by phycocyanin autofluorescence measured at 640 nm. Images were processed with Volocity (PerkinElmer, Waltham, MA) and used to obtain the cell size measurements made along the major and minor axis. Cell volumes were calculated using the equation for an ellipsoid, $V=\frac{4}{3}\pi a^{2}b$, where *a* is the diameter of the minor axis and *b* is the diameter of the major axis.

### RNA isolation and sequencing.

Cells from a steady-state turbidostat cultures were collected via centrifugation at 7,000 rpm for 5 min at 4°C, flash frozen in liquid nitrogen, and stored at −80°C. Total RNA extraction was performed using standard methodology (54). The quality and integrity of the RNA was assessed on an Agilent 2100 bioanalyzer, and only samples with integrity numbers between 8 and 10 were selected. Template cDNA was prepared using the Applied Biosystems SOLiD total RNA-Seq (transcriptome sequencing) kit (Life Technologies, Carlsbad, CA) according to the manufacturer’s protocol. Sequencing was carried out using the SOLiD 5500XL protocol (Life Technologies). The 50-base sequence reads were mapped to the genomes of *Synechococcus* 7002 (GenBank accession number NC_010475), and gene expression levels were determined using the Rockhopper software package as previously described (55). Genes that showed expression values in the bottom 20% of genes at each *I_i_* level for which mRNA was collected were removed from analysis. Hierarchical clustering was performed in the MatLab Bioinformatics Toolbox (MathWorks, Inc.) to identify six distinct profiles of genes with similar expression profiles across irradiance-controlled steady states. Gene expression values (reads per kilobase per million reads [RPKM]) were transformed to log base 2 values and standardized (mean = 0 and standard deviation = 1). Genes in each of the six clusters (profiles) chosen for further analysis were examined to determine whether certain functions were enriched in a given module. Enrichment is defined as the percentage of genes within the profile for which a function has been assigned being significantly higher than the percentage of genes of the same function in the entire genome with a *P* value of <0.05 according to Fisher exact test.

### Photosynthetic performance.

Chl *a* fluorescence measurements were performed on samples obtained from steady-state turbidostat cultures using pulse amplitude-modulated fluorometry (PAM) in a DUAL-PAM-100 instrument equipped with a photodiode detector and RG665 filter (Walz GmbH, Effeltrich, Germany). Samples were dark adapted for 1 min prior to analyses. Red measuring light (620 nm) was pulsed at the lowest power at 1,000 Hz in the dark and at 10,000 Hz during actinic illumination at 98 µmol photons ⋅ m^−2^ ⋅ s^−1^ with 635-nm light. Transient fluorescent changes were measured after fluorescence induction through the following: (i) a 200-ms saturating pulse (2,000 µmol photons ⋅ m^−2^ ⋅ s^− 1^), (ii) 5 s of only far-red light (730 nm), (iii) another 15 s of actinic light, and (iv) 30 s of darkness. The rate of change (rise) of postillumination fluorescence occurring in the dark during step iv results from reduction of plastoquinone (PQ) from NAD(P)H or other reductant accumulated during illumination and was interpreted as a proxy for the rate of cyclic electron transport (rCEF) around PS I (22, 56). Calculations for determining the relative electron transport rates (rETR = PAR × Δ*F*/*F_m_*′) have been previously described (57, 58). Rapid light curves were generated by evaluating rETR as a function of increasing PAR values (1-min step intervals). The maximum values and initial slopes (rETR_max_ and α_r_, respectively) were determined and interpreted as the relative photosynthetic capacity and relative maximal photosynthetic quantum yield of photochemistry, respectively (58).

### Calculations and regression.

The net rate of O_2_ production was calculated from the steady-state mass balance through the bioreactor control volume (equation 1).

(1) $$q_{{\text{O}}_{2}}x=D\left(\left[{\text{O}}_{\text{2}}^{\text{in}}]-[{\text{O}}_{\text{2}}\right]\right)+k_{l}a\left(k_{H}p{\text{O}}_{\text{2}}^{\text{in}}-[{\text{O}}_{\text{2}}]\right)$$

The specific rate of O_2_ production (*q*_O2_) multiplied by the biomass concentration (*x*) is interpreted here as the net rate of O_2_ production during photosynthesis (22) and is a function of the dilution rate (*D*), the lumped mass transfer coefficient (*k_l_a*) (0.83 min^−1^), dissolved O_2_ concentration ([O_2_]), and Henry’s law partitioning coefficient (*k_H_* = 1.08 mM atm^−1^). The specific rate of biomass production (*q_x_*; Cmmol biomass hour^−1^ ⋅ gram_AFDW_^−1^) was calculated by assuming the molecular weight of dry biomass to be 24.59 g_AFDW_ ⋅ C mol^−1^ (59). Parameterization and curve fitting were performed via nonlinear least-squares regression using the fit function in MatLab with a weighted fit option. All saturating and photoinhibition relationships of growth and photosynthesis were fit with equation 2, where the inhibition index (β) was simplified from the form originally reported by Platt et al. (60) to more clearly represent the theoretical irradiance threshold of inhibition; parameters *I_i_* and *I_k_* are the scalar incident and theoretical saturating irradiances, respectively.

(2) $$R=R_{\text{max}}\left[1-\text{exp}\left(\frac{-I_{i}}{I_{k}}\right)\right]\text{exp}\left(\frac{-I_{i}}{\beta }\right)$$

## SUPPLEMENTAL MATERIAL

Figure S1  *In situ* photosynthesis-irradiance curves. Each *P*_net_ value represents the net potential for oxygenic photosynthesis at each corresponding incident irradiance value controlled within the photobioreactor for *Synechococcus* 7002 (○) and *Cyanothece* 51142 (□) steady-state levels corresponding to adaptation to 99 and 211 µmol photons ⋅ m^−2^ ⋅ s^−1^, respectively. Values are means ± 1 standard deviation (error bars). Nonlinear regression was used to estimate *P*_net_ for *Synechococcus* 7002 at higher *I_i_* values (dotted line). Download Figure S1, DOCX file, 0.1 MB

Figure S2  Optimal quantum yield of PS II (*Y*_II_ = *F_v_*/*F_m_*). Data points specific to *Synechococcus* 7002 and *Cyanothece* 51142 are represented by red circles and blue squares, respectively. Linear regression was used to establish positive or negative trends of each parameter during light-limited growth (solid lines), saturated/peak growth (dotted lines), and photoinhibited growth (dashed lines). Note that *F_v_* = (*F_m_* – *F_o_*) and is known and previously reported to be subject to ±100% error in cyanobacteria (Campbell et al., 1998; Schreiber, 2004). Hence, data points and trends are not fully conclusive. Download Figure S2, DOCX file, 0.4 MB

Table S1  All genes that were grouped into clusters I to IV. Expression levels under all conditions examined in this study and annotation data are also shown. Table S1, XLSX file, 0.5 MB

Table S2  All genes that were grouped into cluster I. Expression levels under all conditions examined in this study and annotation data are also shown. Table S2, XLSX file, 0.3 MB

Table S3  All genes that were grouped into cluster II. Expression levels under all conditions examined in this study and annotation data are also shown. Table S3, XLSX file, 0.3 MB

Table S4  All genes that were grouped into cluster III. Expression levels under all conditions examined in this study and annotation data are also shown. Table S4, XLSX file, 0.2 MB

Table S5  All genes that were grouped into cluster IV. Expression levels under all conditions examined in this study and annotation data are also shown. Table S5, XLSX file, 0.2 MB
