# Supplementary material for: Unlocking the Constraints of Cyanobacterial Productivity: Acclimations Enabling Ultrafast Growth
Source: mBio. 2016 Jul 26;7(4):e00949-16. doi: 10.1128/mBio.00949-16 (PMC4981716; doi:10.1128/mBio.00949-16)
Supplement: Figure S1 — In situ photosynthesis-irradiance curves. Each Pnet value represents the net potential for oxygenic photosynthesis at each corresponding incident irradiance value controlled within the photobioreactor for Synechococcus 7002 (○) and Cyanothece 51142 (□) steady-state levels corresponding to adaptation to 99 and 211 µmol photons ⋅ m−2 ⋅ s−1, respectively. Values are means ± 1 standard deviation (error bars). Nonlinear regression was used to estimate Pnet for Synechococcus 7002 at higher Ii values (dotted line). Download [file mbo003162888sf1.docx]

**
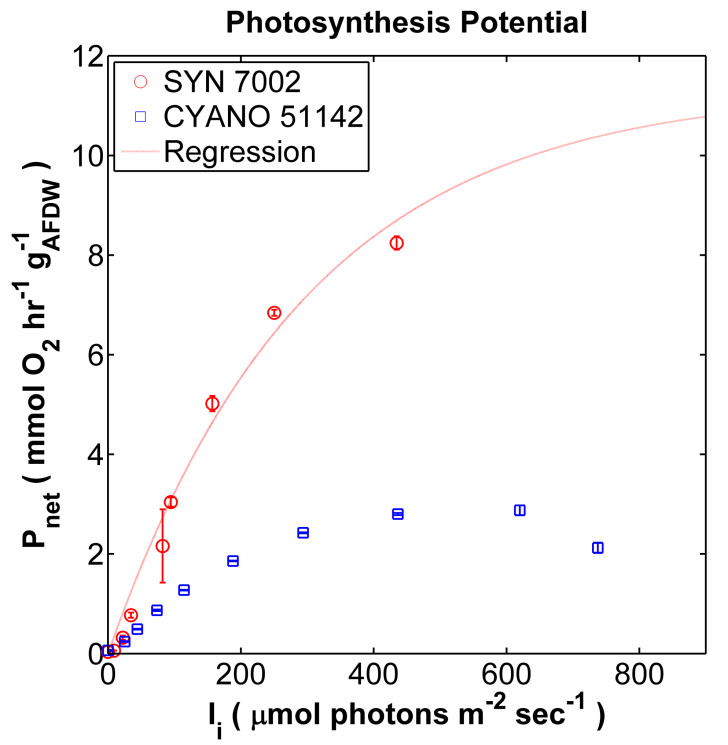
**

**Figure S1.** *In situ* photosynthesis-irradiance curves. Each P_net_ value represents the net potential for oxygenic photosynthesis at each corresponding incident irradiance value controlled within the photobioreactor for: *Synechococcus* 7002 [○] and *Cyanothece* 51142 [□] steady states corresponding adapted to 99 and 211 µmol photons·m^-2^·sec^-1^, respectively. Error bars on data point symbols represent ± 1 standard deviation. Non-linear regression was used to estimate P_net_ for *Synechococcus* 7002 at higher I_i_ values (dotted line).
