# Supplementary material for: Unlocking the Constraints of Cyanobacterial Productivity: Acclimations Enabling Ultrafast Growth
Source: mBio. 2016 Jul 26;7(4):e00949-16. doi: 10.1128/mBio.00949-16 (PMC4981716; doi:10.1128/mBio.00949-16)
Supplement: Figure S2 — Optimal quantum yield of PS II (YII = Fv/Fm). Data points specific to Synechococcus 7002 and Cyanothece 51142 are represented by red circles and blue squares, respectively. Linear regression was used to establish positive or negative trends of each parameter during light-limited growth (solid lines), saturated/peak growth (dotted lines), and photoinhibited growth (dashed lines). Note that Fv = (Fm – Fo) and is known and previously reported to be subject to ±100% error in cyanobacteria (Campbell et al., 1998; Schreiber, 2004). Hence, data points and trends are not fully conclusive. Download [file mbo003162888sf2.docx]

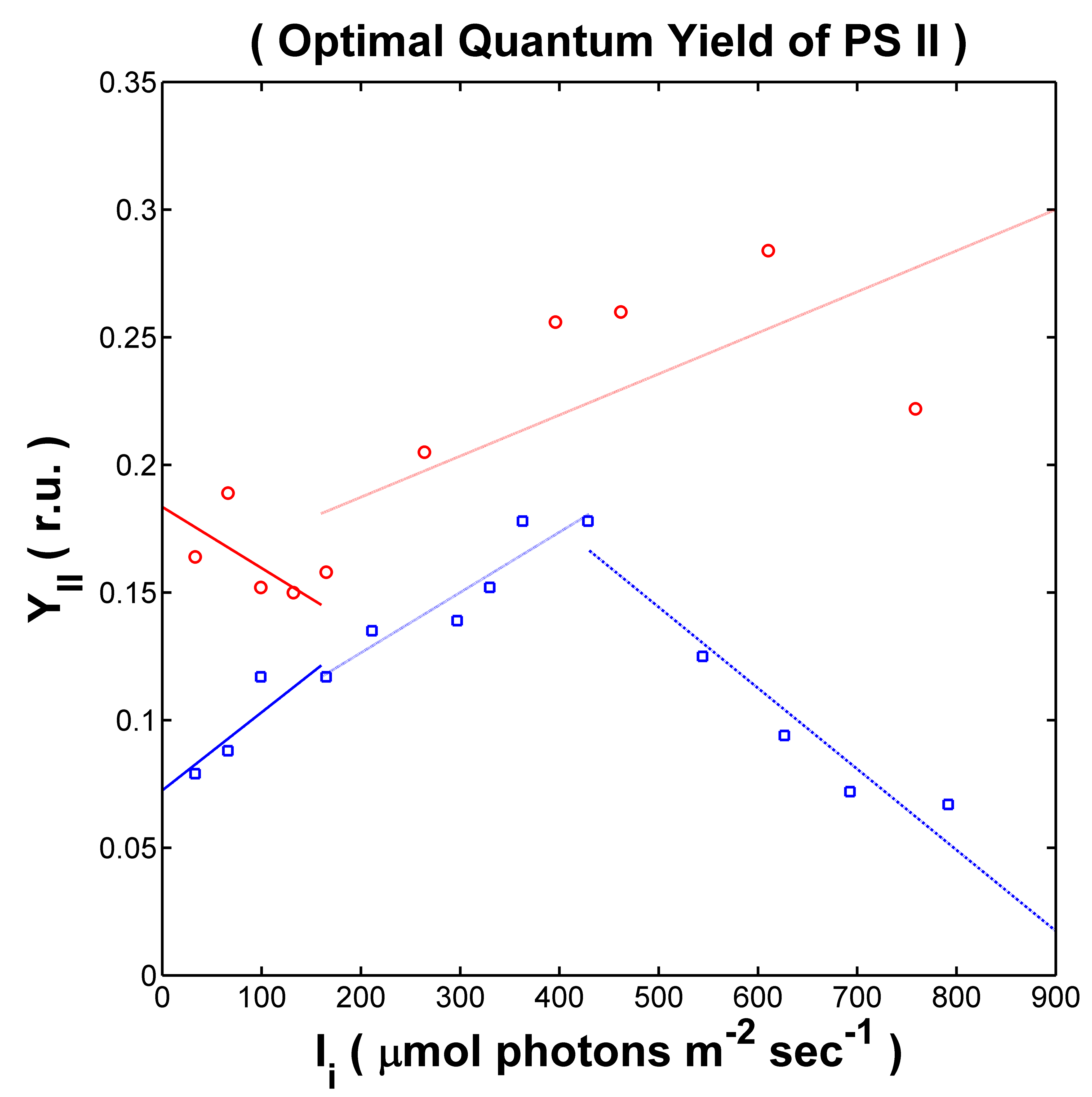


**Figure S2.** Optimal quantum yield of PS II (Y_II_ = F_v_/F_m_). Data point specific to *Synechococcus* 7002 and Cyanothece 51142 are represented by [○] and [□] symbols, respectively. Linear regression was used to establish positive or negative trends of each parameter during: light-limited growth (solid lines), saturated/peak growth (dotted lines) and photo-inhibited growth (dashed lines). Note that F_v_ = (F_m_ – F_o_) and is known and previously reported to be subject to ± 100% error in cyanobacteria (Campbell et al., 1998;Schreiber, 2004). Hence, data points and trends are not fully conclusive,
